# Intermittency and Critical Scaling in Annular Couette Flow

**DOI:** 10.3390/e22090988

**Published:** 2020-09-04

**Authors:** Kazuki Takeda, Yohann Duguet, Takahiro Tsukahara

**Affiliations:** 1 Department of Mechanical Engineering, Tokyo University of Science, Chiba 278-8510, Japan; tairyuuuu88@gmail.com; 2 LIMSI-CNRS, Université Paris-Saclay, F-91400 Orsay, France; duguet@limsi.fr

**Keywords:** subcritical phenomenon, transition to turbulence, direct numerical simulation

## Abstract

The onset of turbulence in subcritical shear flows is one of the most puzzling manifestations of critical phenomena in fluid dynamics. The present study focuses on the Couette flow inside an infinitely long annular geometry where the inner rod moves with constant velocity and entrains fluid, by means of direct numerical simulation. Although for a radius ratio close to unity the system is similar to plane Couette flow, a qualitatively novel regime is identified for small radius ratio, featuring no oblique bands. An analysis of finite-size effects is carried out based on an artificial increase of the perimeter. Statistics of the turbulent fraction and of the laminar gap distributions are shown both with and without such confinement effects. For the wider domains, they display a cross-over from exponential to algebraic scaling. The data suggest that the onset of the original regime is consistent with the dynamics of one-dimensional directed percolation at onset, yet with additional frustration due to azimuthal confinement effects.

## 1. Introduction

The dynamics at the onset of turbulent fluid flow, as the parameters are varied, is one of the most puzzling issues of hydrodynamics. Subcritical flows are known to feature two regimes in competition, namely a laminar and a turbulent one. As the Reynolds number (their main control parameter) is varied, this competition takes the form of laminar-turbulent coexistence featuring some interesting analogies with phase transitions in thermodynamics. The onset of this coexistence in wall-bounded shear flows has been speculated to follow a statistical scenario called directed percolation (DP). It involves a critical point (a critical Reynolds number) in the vicinity of which fluctuations diverge algebraically [1,2]. The directed percolation scenario has gained theoretical importance because it appears as the usual rule for a one-dimensional systems obeying a set of specific properties, notably a unique absorbing state and short-range interactions [3,4]. However, it quickly proved difficult to isolate similar phenomena experimentally [5]. The main limitations happen to be finite-size effects, as well as the presence of defects [6,7,8] or issues revolving around nucleation rates [9,10]. The first experimental evidence for directed percolation in a two-dimensional physical system, with a complete set of critical exponents, occurred in electroconvection in nematic liquid crystals [11]. More recent experiments and numerical simulations with inert liquids were aimed at establishing the critical exponents relevant for the laminar-turbulent transition. The only meaningful experimental results are to be found in Ref. [12] for the flow inside an annulus driven by the revolutions of the outer wall, where all critical exponents match those of (1 + 1)-D DP. All other experimental attempts in effectively two-dimensional geometries have so far lead to ambiguous results [13,14]. A few numerical studies based on other geometries have also confirmed the DP hypothesis in one dimension, among them [15]. The most notorious system displaying one-dimensional spatiotemporal intermittency (STI) is cylindrical pipe flow. Although (1 + 1)-D DP has been widely speculated and is found in the most recent modelling approaches [16,17,18], clean experimental evidence seems to require facilities of a size beyond anything engineerable [19]. The only convincing two-dimensional study to date based on the (underesolved) Navier–Stokes equations and supporting the DP hypothesis is found in Ref. [20]. There again, a cost compromise was necessary between accuracy of the Navier–Stokes solutions and size effects. There the set of critical exponents differs from their unidimensional counterpart and corresponds to (2 + 1)-D DP. The status of the application of (2 + 1)-D DP to other planar flows is still open: for plane Couette flow (pCf), finite-size effects wrongly predict to discontinuous scenarios [21], whereas plane Poiseuille flow (pPf) seems to display a two-stage behavior so far poorly understood [22,23,24]. At a finite distance from the critical point, these two planar flows feature more structured arrays of turbulent stripes, all oblique to the mean flow direction (see, e.g., [21,25,26,27,28,29,30] for recent reviews).

Given the current status of DP affairs in shear flows, new flow candidates where to probe the DP hypothesis are encouraged, irrespective of the effective dimension considered (one or two). In the present article, we revisit transition in annular Couette flow (aCf) in the light of critical scaling. This flow has a geometry similar to cylindrical pipe flow, however, with a solid cylinder at its centerline. The geometry is determined by the radius ratio η between the radius of the outer pipe and that of the inner one. This flow supports both turbulence [31] as well as a linearly stable base flow for all Reynolds number of interest, hence transition has to be of the subcritical type. Unlike annular pipe flow [32,33,34], no pressure gradient is applied, instead the fluid is entrained by the translating motion of the inner cylinder [35]. Earlier work by some of us [36] on this flow have lead to surprising results: although the transitional flow reported for η≥0.5 consists of helical bands of turbulence wrapping around the inner rod, for lower values of η, a new regime of laminar-turbulent alternations was reported. This regime is characterized by slightly shorter streamwise correlations and non-oblique structures, explained by the azimuthal confinement and by the impossibility to host azimuthal large-scale flows [37]. The aim of the present article is to give a more detailed characterization of the novel low-η intermittent regime and of its onset. In particular, the azimuthal extension of aCf is investigated in a range of parameters beyond that used by Kunii et al. [36]. As will be seen, this new choice of geometrical parameters leads to new conclusions regarding the critical exponents. This new parametric study allows one to rationalize once and for all the quantitative comparison between original geometry and the extended one.

The plan of this article unfolds as follows: the geometry and the numerical methods are explained in Section 2, and the statistics of STI are reported in Section 3 and discussed in Section 4.

## 2. Set-Up and Methodology

### 2.1. Geometry of aCf

**Figure 1:**
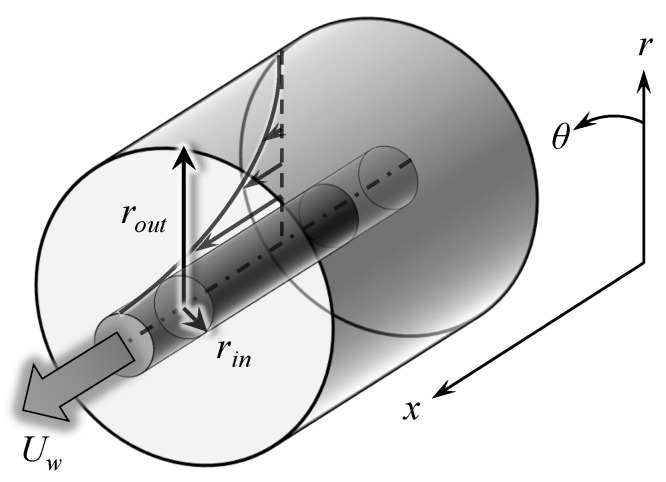
Sketch of annular Couette flow in the cylindrical coordinate system.

Annular Couette flow is the flow in the interstice between two coaxial cylinders of formally infinite length, driven by the motion at velocity $U_{w}>0$ of the inner cylinder in the *x*-direction. The annular geometry of this flow is common to both Taylor–Couette flow and annular Pipe flow; however, the forcing is different and no spin of the walls is considered. A sketch of that geometry is displayed in Figure 1 with the usual notations for the cylindrical coordinates (x,r,θ). Assuming that the inner and outer cylinder have respective dimensional radii $r_{in}$ and $r_{out}$, the main geometrical parameter of this study is the radius ratio $\eta =r_{in}/r_{out}$, which varies in the open interval (0,1). We also introduce the gap *h* between the two cylinders $h=r_{out}-r_{in}$.

Computationally, the pipes require to have either finite length or to be spatially periodic. The use of a spectral Fourier-based method to solve the pressure Poisson equation requires axial and azimuthal periodicity. This introduces the two wavelengths $L_{x}$ and $L_{\theta }$, respectively, as the domain length and the angular periodicity. While $L_{x}$ is a free parameter, the natural value for $L_{\theta }$ is 2π because of the cylindrical geometry. However, there is no computational obstruction to choosing other values for $L_{\theta }$, for instance $L_{\theta }=8\pi$ or 16π as in Ref. [36]. In what follows, we keep the generic notation $L_{\theta }$.

**Figure 2:**
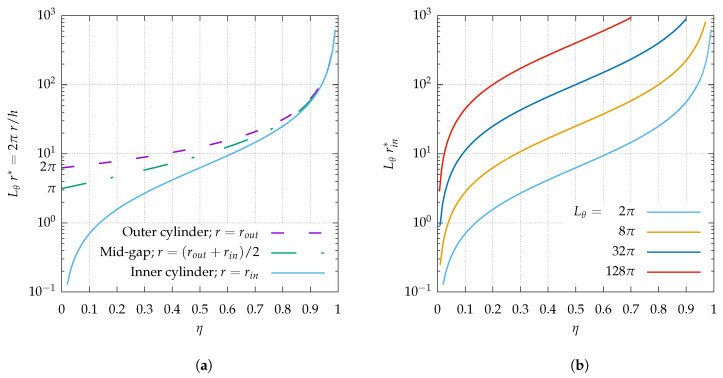
(**a**) circumference of original annular pipe system at the outer cylinder, at mid-gap, and at the inner cylinder; (**b**) circumference at the inner cylinder for $L_{\theta }\geq 2\pi$.

Like in other wall-bounded shear flows, the main lengthscale ruling out the transitional dynamics at onset is the gap *h* between the two solid walls, which here depends directly on the value η via the relation $h=r_{out}\left(1-\eta \right)$. The perimeter on the internal cylinder, at mid-gap or on the external cylinder, now expressed in units of *h*, is shown in Figure 2 when the original dimensional value of $L_{\theta }$ is 2π (Figure 2a). The inner perimeter is also displayed when $L_{\theta }$ is a multiple of 2π (Figure 2b), with $L_{\theta }=2\pi n$. The theory developed in Refs. [33,36] shows that azimuthal large-scale flows cannot be accommodated by the geometry unless $L_{\theta }r/h\gg 1$ everywhere in the domain. The data for the inner cylinder play the role of a lower bound. For $L_{\theta }=2\pi$, it is clear from Figure 2a that, for the lowest values of η, no azimuthal large-scale flow is possible. However, increasing *n* leads to azimuthal large-scale flows being possible for smaller and smaller values of η. This leads to the possibility to artificially restore large-scale flows otherwise ruled out by geometrical confinement.

### 2.2. Governing Equations and Computational Methods

Whereas η is a geometrical parameter only, we also introduce the Reynolds number $Re_{w}=U_{w}h/4\nu$, based on the half velocity of the cylinder sliding $U_{w}/2$, the half gap width h/2, and the kinematic viscosity ν of the fluid. The reason why half-gap and half-velocities are considered to non-dimensionalize the equations is a simple way to reconnect with the standard conventions for pCf as η goes towards unity. By choosing this convention for all values of η, the non-dimensional incompressible equations ruling the flow dynamics without any turbulence model read

(1) $$\nabla^{*}\cdot u^{*}=0,$$

(2) $$\frac{\partial u^{*}}{\partial t^{*}}+\left(u^{*}\cdot \nabla^{*}\right)u^{*}=-\nabla^{*}p^{*}+\frac{1}{4Re_{w}}\Delta^{*}u^{*},$$

where superscripts * indicate quantities non-dimensionalized with $U_{w}$ and *h*, and where $u=(u_{x},\;u_{r},\;u_{\theta })$ and *p* represent the velocity field and the pressure field, respectively.

Equation (2) is discretized in space using finite differences and with fine enough grid resolutions according to the standard criteria of direct numerical simulation (DNS) [26]. The time discretization is carried out using a second-order Crank–Nicolson scheme, and an Adams–Bashforth scheme for the wall-normal viscous term and the other terms, respectively. Further details about the numerical methods used here can be found in Ref. [38]. Table 1 lists the parameters used in this computational study.

## 3. Statistics at the Onset of Transition

### 3.1. Global Stability and Coherent Structures Close to Onset

**Figure 3:**
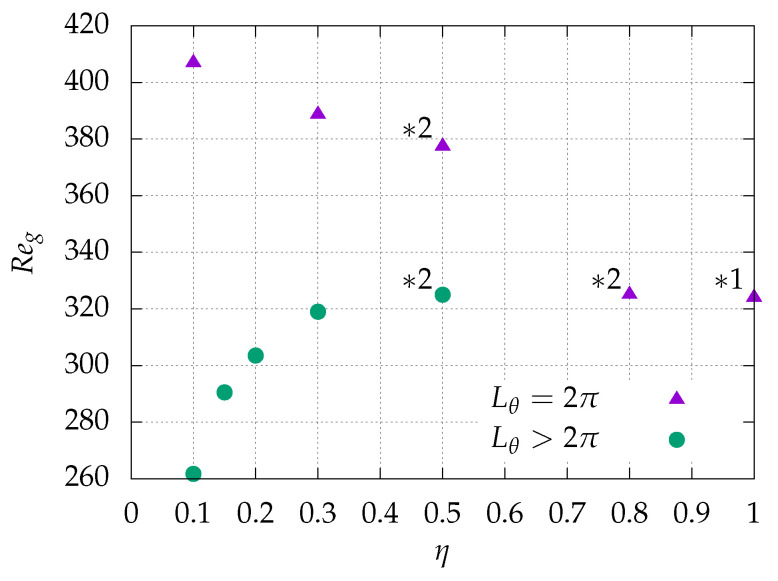
Radius ratio η dependency of the global critical Reynolds number Reg. The plot includes the pCf limit η→1 from Ref. [21] (labeled “*1”), as well as DNS data from Ref. [36] for η=0.5 and 0.8 (labeled “*2”). Triangles: original aCf with $L_{\theta }=2\pi$ is plotted using triangles; circles: artificially extended aCf ($L_{\theta }>2\pi$).

In the present subsection, we recall some key results of Ref. [36] together with some updated predictions. The investigation of the onset of turbulence starts with the determination of the global Reynolds number $Re_{g}$, defined as the highest Reynolds number below which no turbulence can survive (at least in the thermodynamic limit, i.e., over infinite observation times in unbounded domains). Since the flow is subcritical, using a given type of initial condition for this task can lead to overestimates of $Re_{g}$. The commonly adopted strategy, both in experiments and numerics, is that of an adiabatic descent [39] initiated from a turbulent state at sufficiently high Reynolds number. In the limit where the waiting time between successive diminutions of Re is sufficient long, the value at which turbulence gets extinct is a good approximation of $Re_{g}$. Figure 3 displays information about $Re_{g}$ depending on the radius ratio η. For $L_{\theta }=2\pi$ (n=1), $Re_{g}$ increases monotonically with decreasing η. For larger $L_{\theta }$, $Re_{g}$ is always smaller than for the case with $L_{\theta }=2\pi$ and the same value of η, with a now decreasing trend for $Re_{g}\left(\eta \right)$ which is even more marked once η≤0.3. The values of $L_{\theta }$ needed to obtain this curve robustly are all listed in Table 1. As for the case of artificially extended aCf at η=0.1, the result for $L_{\theta }=128\pi$ is plotted in the figure. The parameter range strictly below η=0.1 has not been investigated.

**Figure 4:**
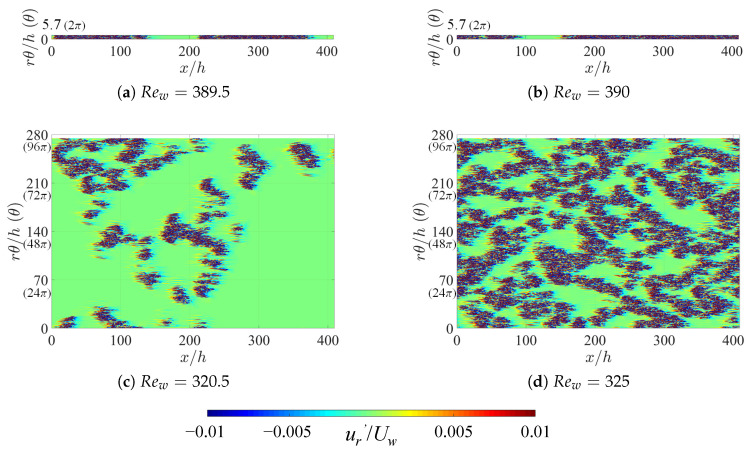
Contours of radial velocity fluctuations $u_{r}^{*}$ at mid-gap for η=0.3 around $Re_{w}=Re_{g}$. Typical snapshots of instantaneous flow fields obtained after reaching each equilibrium state are shown here. The main flow is from left to right. (**a**,**b**) original aCf with $L_{\theta }=2\pi$, and (**c**,**d**) artificially extended with $L_{\theta }=96\pi$.

**Figure 5:**
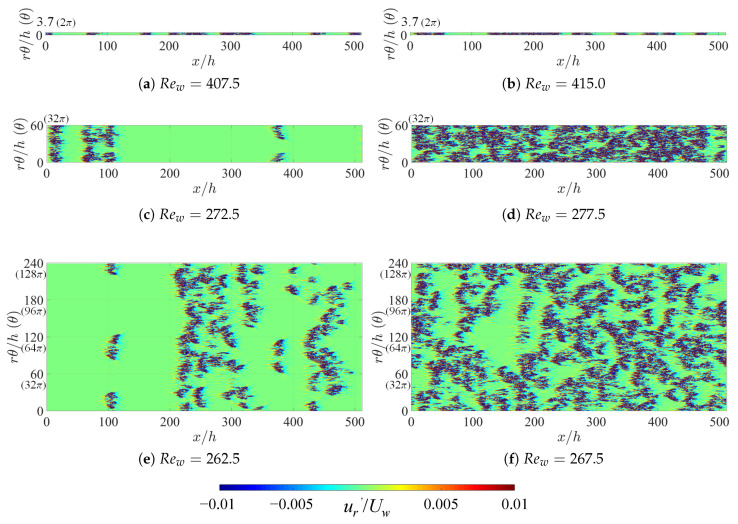
The same as Figure 4, but for η=0.1. (**a**,**b**) $L_{\theta }=2\pi$; (**c**,**d**) $L_{\theta }=32\pi$; and (**e**,**f**) $L_{\theta }=128\pi$.

The fact that artificially extended systems display a lower threshold in Re indicates that some specific spatiotemporal regimes, specific to large $L_{\theta }$ and not allowed for in narrow domains, are able to maintain themselves against relaminarization. As in Ref. [36], we can compare typical snapshots of the velocity fields in the corresponding regime in order to highlight the qualitative differences. Figure 4 and Figure 5 display instantaneous snapshots of the radial velocity at mid-gap (i.e., $r=(r_{in}+r_{out})/2$) at respectively η=0.3 and 0.1, one very close to $Re_{g}$ (left column) and the other slightly above it (right column). Each row corresponds to a different value of the integer *n* (*n* = 1, 16, 48, and 64), i.e., another value of $L_{\theta }$. When n=1, the one-dimensional intermittency is reminiscent of the dynamics in cylindrical pipe flow [40]. The differences between different values of η emerge only for higher *n*. For η=0.3, the stripe patterns exhibit an obliqueness typical of most laminar-turbulent patterns [25,26,37,41]. However, it is visually clear that the situation is different for η=0.1, with shorter structures and less pronounced obliqueness. It is not immediately clear whether the effective dimensionality of the proliferation process is rather one or two. These issues can be addressed using the determination of critical exponents, as will be done in the next subsection.

### 3.2. Data Binarization

Velocity fluctuations with respect to the mean flow are defined as $u^{{}^{\prime }}=u-\bar{u}$, where $\bar{u}$ is the space-averaged time-dependent velocity averaged along *x* and θ, as defined in Equation (3). Here, *y* denotes the (dimensional) distance from the inner cylinder to the outer cylinder as $y=r-r_{in}$, instead of using *r*.

(3) $$\bar{u}\left(y,t\right)=\frac{1}{L_{x}L_{\theta }}\int_{0}^{L_{x}}\int_{0}^{L_{\theta }}u\left(x,y,\theta ,t\right)dxd\theta .$$

The flow is separated into its laminar and turbulent components by postulating a threshold independently of the Reynolds number. The local criterion chosen is $|u_{r}^{\prime }/U_{w}|\geq 0.01$ for turbulence and $|u_{r}^{\prime }/U_{w}|<0.01$ for laminar flow, with $u_{r}^{\prime }$ the radial velocity component, which vanishes everywhere for strictly laminar flow. As in Figure 4 and Figure 5, localized turbulent regions are visualized by contours of ${u_{r}^{\prime }}^{*}$ in steps of ±0.01. The turbulent fraction $F_{t}$ is evaluated at mid-gap (y=h/2) by estimating the percentage of grid points for which the turbulent criterion above is fulfilled.

**Figure 6:**
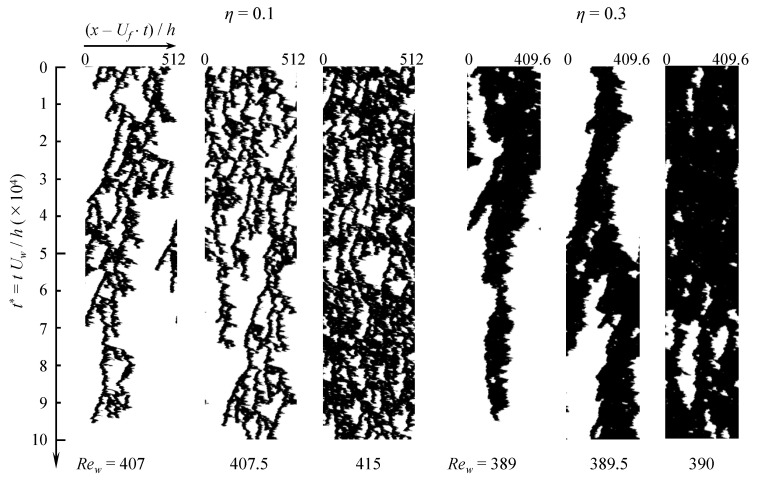
Space-time $\left(x-U_{f}t\right)$ diagram of original aCf ($L_{\theta }=2\pi$) for η=0.1 (three leftmost columns) and 0.3 (three rightmost columns). Black: turbulence according to the criterion ${\langle {u_{r}^{\prime }}_{\mathrm{rms}}\rangle }_{\theta }/U_{w}\geq 0.01$. The values of the frame velocity $U_{f}$ for η=0.1 are $0.288U_{w}$ at $Re_{w}=407$, $0.2875U_{w}$ at $Re_{w}=407.5$, $0.2815U_{w}$ at $Re_{w}=415$, and those for η=0.3 are approximately equal to $\bar{u_{m}}$.

The dynamics of the proliferation process for η=0.1 and 0.3 is illustrated in Figure 6 using space-time diagrams and compared one to another in the case n=1. The spatial variable is $x-U_{f}t$, i.e., the streamwise coordinate in a frame moving with constant velocity $U_{f}$, which is close to the average velocity $\bar{u_{m}}$. The space-time diagram is based on the binarized radial velocity $u_{r}^{\prime }$. The absolute value of the radial velocity evaluated at mid-gap is first averaged azimuthally according to

(4) $${\langle {u_{r}^{\prime }}_{\mathrm{rms}}\rangle }_{\theta }\left(x,t\right)=\sqrt{\frac{1}{2\pi }\int_{0}^{2\pi }u_{r}^{\prime }{}^{2}\left(x,h/2,\theta ,t\right)\;d\theta .}$$

and the binarization criterion is ${\langle {u_{r}^{\prime }}_{\mathrm{rms}}\rangle }_{\theta }/U_{w}\geq 0.01$. The frame velocity $U_{f}$ for η=0.3 is chosen to be same with $\bar{u_{m}}$, which is estimated in two steps. First, a spatially average velocity is evaluated at every time *t*

(5) $$u_{m}\left(t\right)=\frac{1}{L_{x}\left(r_{out}^{2}-r_{in}^{2}\right)L_{\theta }}\int_{0}^{L_{x}}\int_{r_{in}}^{r_{out}}\int_{0}^{L_{\theta }}u_{x}\left(x,y,\theta ,t\right)\;rdxdrd\theta ,$$

then it is time-averaged using a classical moving average technique over a time interval ΔT (with $\Delta T>10^{4}h/U_{w}$ after reaching equilibrium).

(6) $$\bar{u_{m}}=\frac{1}{\Delta T}\int_{T}^{T+\Delta T}u_{m}\left(t\right)dt.$$

We found that, for η=0.1, an optimal value of $U_{f}$ for the frame to move with puffs was slightly slower than $\bar{u_{m}}$. For each value of η, three space-time diagrams are displayed, respectively below, close to and above the corresponding critical point $Re_{g}\left(\eta \right)$. The shorter aspect of the coherent structures for η=0.1 is striking compared to η=0.3. Many more splitting and decay events, qualitatively similar to the pipe flow case [40,42,43], occur for η=0.1 despite equal pipe lengths. This suggests that the status of the present simulations for η=0.1 is qualitatively much closer to the thermodynamic limit than it is for η=0.3. As a by-product, the critical scaling is expected to converge at a lower price than at higher η. Given the cost obstacles induced by the diverging lengthscales/timescales in most critical phenomena, the above conclusion is positive news.

### 3.3. Intermittency Statistics

The statistical post-processing protocol for STI is vastly similar to that used by other authors: the first step is to monitor the decay in the time of the turbulence fraction $F_{t}\left(t\right)$ when the system is initiated with turbulence everywhere. By dichotomy, this yields a good approximation of $Re_{g}$ and allows one to define the reduced control parameter $\varepsilon =\left(Re_{w}-Re_{g}\right)/Re_{g}$. This decay is expected to be algebraic exactly at onset, i.e., of the form $F_{t}\left(t\right)=O\left(t^{-\alpha }\right)$. This yields as well the so-called dynamic exponent α. In a second phase, the equilibrium turbulent fraction (i.e., its time average) is monitored as a function of ε. For ε>0, the data *versus* the expected scaling $F_{t}\left(t\right)=O\left(\varepsilon^{\beta }\right)$ yield the exponent β. Eventually, the mean correlation length $\xi (Re_{w})$ (either $\xi_{x}$ in the streamwise direction or $\xi_{\theta }$ in the azimuthal one) can be estimated at equilibrium by monitoring the cumulative distribution function (CDF) of the laminar gaps $P_{lam}\left(l_{x}>L\right)$, where $l_{x}$ stands for the length of a laminar trough and *L* is a dummy variable. A critical exponent $\mu_{⊥}$ can be evaluated from fits as the algebraic decay exponent of the CDF.

**Figure 7:**
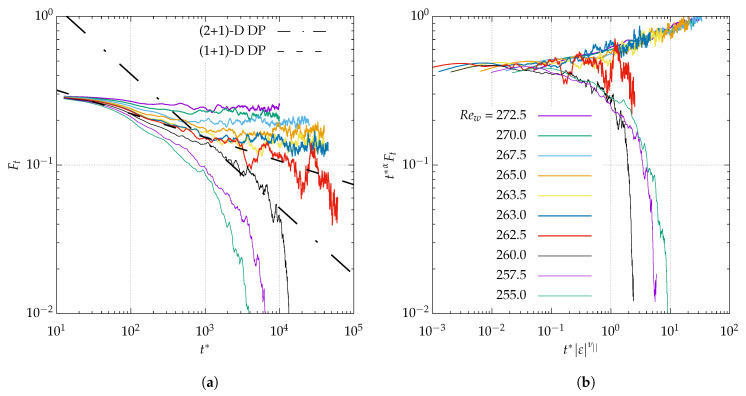
Critical quenches from $Re_{w}=280$ to each Reynolds number. Temporal variation of turbulent fraction $F_{t}$ for η=0.1 and $L_{\theta }=128\pi$ (log-log scale). In (**a**), the black dashed-dotted line and dashed line each indicate possible algebraic fits with the dynamic exponent α from (2 + 1)-D and (1 + 1)-D directed percolation (respectively α = 0.451 and 0.159). See also Supplementary; (**b**) test of the 1D scaling hypothesis by plotting $t^{\alpha }F_{t}$ vs. $t\varepsilon^{\nu_{\left|\right|}}$ (log-log scale), with $\nu_{\left|\right|}$=1.733 for (1 + 1)-D DP.

We begin by describing the results from the critical quench experiments of Figure 7 for η=0.1 and n=64. The initial condition corresponds to a turbulent velocity field from a long simulation well above $Re_{g}$, here taken as $Re_{w}=280$. The same initial condition is used for new simulations at another target value of $Re_{w}$, in principle such that $Re_{w}$ is “close” to $Re_{g}$. As expected, the flow relaminarizes (attested by the monotonic decrease of $F_{t}\left(t\right)$) for sufficiently low values of $Re_{w}$, whereas it stays turbulent for the higher values. In the latter case, the turbulent fraction reaches a non-zero mean value $\bar{F_{t}}$, which will be reported in the next figure. The set of colored curves in Figure 7a straddle the decay curve corresponding to the critical value $Re_{w}=Re_{g}$, whose best approximation in the figure is the red curve associated with $Re_{w}=262.5$. For continuous phase transitions, the corresponding decay is expected to be of power-law type, i.e., $F_{t}=O\left(t^{-\alpha }\right)$. This fact of $260<Re_{g}<262.5$ yields an approximation of $Re_{g}=261.7$, which allows for defining ε as before. The present approach rests on the hypothesis of a critical scaling in the vicinity of the critical point. If that hypothesis is correct then, by rescaling time and turbulent fraction, the curves of Figure 7a should collapse onto two master curves, one for the relaminarization process and the other for the saturation process. This is tested in Figure 7b by plotting $t^{\alpha }F_{t}\left(t\right)$ as a function of the rescaled time ${t|\varepsilon |}^{\nu_{\|}}$. As for α and $\nu_{\|}$, the approximate values from (1 + 1)-D DP theory, respectively 0.451 and 1.733, have been used for the rescaling. The match is satisfying, which confirms that a critical range has been identified in this system.

**Figure 8:**
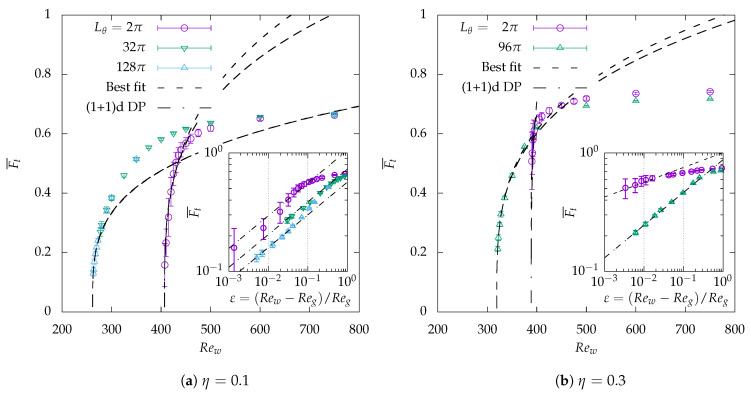
Reynolds-number dependence of the time-averaged turbulent fraction $\bar{F_{t}}$ vs $Re_{w}$ for the different radius ratios in the original domain ($L_{\theta }=2\pi$) and in artificially extended domains ($L_{\theta }\gg 2\pi$). Vertical error bars: standard deviations of $F_{t}$ during the averaging period. Dashed/dashed-dotted line: algebraic fits $\bar{F_{t}}=O\left(\varepsilon^{\beta }\right)$, with exponent β obtained either as best fit $\beta_{\mathrm{fit}}$ or from the (1 + 1)-D DP universality class $\beta_{1D}=0.276$. In each figure, the insets are plotted in log-log coordinates *versus* ε that is determined with $Re_{g}$ presented in Table 2.

As a by-product of Figure 7, the values of the mean turbulent fraction $\bar{F_{t}}$, obtained after reaching equilibrium, are reported in Figure 8 as functions of $Re_{w}$. Critical theories all predict a scaling $\bar{F_{t}}=O\left(\varepsilon^{\beta }\right)$ close enough to the critical point. The algebraic scaling revealed in the previous plots of critical quench suggests that, for instance, Re=262.5 belongs to the range where algebraic fits apply for η=0.1 and $L_{\theta }=128\pi$. Consequently, if, for these parameters, ε is defined using the approximated $Re_{g}=261.7$, the dependence of $\bar{F_{t}}$ *versus* ε is also expected to be algebraic in the same range of values of Re. In that case, the power-law exponents can be classically estimated using log-log plots and compared to those from DP theories. Algebraic fits of $\bar{F_{t}}$ are shown in Figure 8 both for η=0.1 (left) and 0.3 (right). For each case, the main plot of $\bar{F_{t}}$ *versus* $Re_{w}$ is displayed in linear coordinates, while the inset displays $\bar{F_{t}}$ *versus* ε in log-log coordinates, in order to highlight the quality of the estimation of the power-law exponent.

The details of the fitting procedure for the various parameters used are given in Table 2. It includes the values of the best fitted exponents as well as the approximate fitting range. As could already be deduced graphically from the insets in Figure 8a, for η=0.1, the compatibility of the exponent β with the theoretical value of $\beta_{1D}=0.276$ from (1 + 1)-D DP is good (to the second digit). This is confirmed for both η=32π and η=128π, which suggests that the thermodynamic limit is already reached, at least as far as the determination of the exponent β is concerned. For $L_{\theta }=2\pi$ the approximated exponent is 0.31 which constitutes a less accurate, but still consistent approximation of the theoretical exponent. For $L_{\theta }=2\pi$, the range of validity of the algebraic fits extends up to ≈5%, whereas it exceeds 10% for $L_{\theta }\geq 32\pi$. For η=0.3, the situation is slightly different: for a large azimuthal extent $L_{\theta }=96\pi$, there is a very good match with the 1D theoretical exponent all the way up to ε≈20%. For $L_{\theta }=2\pi$, however, although an algebraic fit seems consistent with the data below ε<1% the measured exponent is closer to 0.12 than to 0.276: none of these values matches any of the percolation theories.

The interpretation is delicate. On one hand, algebraic fits seem always verified as soon as ε is small enough; on the other hand, (1 + 1)-D percolation exponents are well approximated only for sufficient azimuthal extension of the order of 100π or more. The original system with $L_{\theta }=2\pi$ hence needs to be interpreted as a system with the DP property that experiences a *geometrical frustration* due to lateral confinement. The present data support the hypothesis that the frustration effect is stronger for η=0.3 than for η=0.1, and thus that the quality of the DP fit will be correspondingly worse. Conversely, the convergence towards the thermodynamic limit seems slower for larger η.

Importantly, we emphasize the main difference between the present conclusion and that by Kunii et al. [36], where the azimuthal extension for η=0.1 was limited to $L_{\theta }=16\pi$ (to be compared to the present values of 32π and 128π). The fits reported in Figure 16 of that article suggested a fit compatible with the (2 + 1)-D exponent $\beta_{2D}=0.583$. This former result, in the light of the present computations, is re-interpreted now as a finite-size effect.

**Figure 9:**
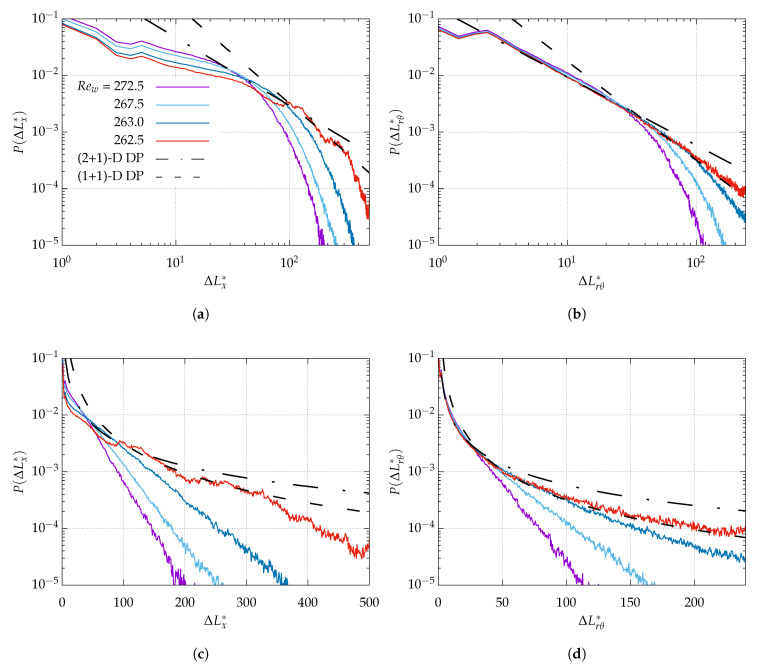
Time-averaged distributions of laminar gap in (**a**,**c**) the streamwise direction and (**b**,**d**) the azimuthal direction, evaluated at mid-gap. (**a**,**b**) log-log plots vs. (**c**,**d**) lin-log plots. $L_{\theta }=128\pi$, η=0.1 as in Figure 5e,f. In both figures, black dashed-dotted line (- · -) and dashed lien (- - -) indicate theoretical distributions $P\left(\Delta L^{*}\right)∼{\Delta L^{*}}^{-\mu_{⊥}}$ with exponents $\mu_{⊥}$ from the universality classes of (2 + 1)-D DP and (1 + 1)-D DP, respectively, i.e., ${\mu_{⊥}}_{2D}=1.84$, and ${\mu_{⊥}}_{1D}=1.748$.

A power-law dependence of $\bar{F_{t}}$ alone does not warrant the proximity to the critical point, as pointed out by Shimizu and Manneville [23] for pPf. Although the critical quenches reported earlier also suggest power-law statistics near the picked up values for $Re_{g}$, the classical determination relies on, at least, three independent algebraic exponents. In order to lift this ambiguity, we chose to report in Figure 9 statistics of laminar gap size for different values of $Re_{w}$ near the suspected critical point. Expecting possible anisotropy when the domain is artificially extended in θ, two kinds of statistics have been monitored, similarly to the study of Chantry et al. [20]. The axial extent of the gaps for η=0.1 and $L_{\theta }=128\pi$ is shown in Figure 9a in log-log coordinates (and Figure 9c in lin-log representation). The azimuthal extent of the laminar gaps is shown in Figure 9b in log-log coordinates (and Figure 9d in lin-log representation). All four figures support a cross-over from exponential to power-law statistics as $Re_{w}$ approaches the value of 262.5, with a decay exponent graphically compatible with the decay exponent $\mu_{⊥}$ of (1 + 1)-D DP. The cross-over appears, however, more clearly in the azimuthal where the match with the theoretical value of $\mu_{⊥}$ is valid over a full decade. In the streamwise direction, the trend is not clear enough to extract a critical exponent with full accuracy. This confirms, however, that the present statistics are indeed gathered in a relevant neighborhood of the critical point and that, for these parameters, $Re_{w}=262.5$ is a decent working approximation of $Re_{g}$.

### 3.4. Dynamics of Localized Turbulent Patches

**Figure 10:**
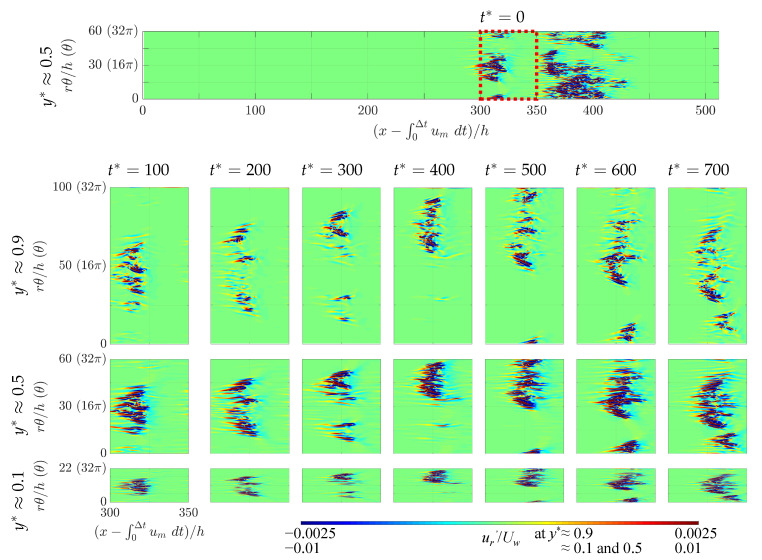
Snapshots of splitting and self-colliding events in aCf for $Re_{w}=262.5$ with $L_{\theta }=32\pi$ and η=0.1. Radial velocity in a frame moving with bulk velocity $\bar{u_{m}}$. Here, t=0 is an arbitrary time instant after reaching equilibrium. Top row, $y^{*}=y/h\approx 0.9$; center row, $y^{*}\approx 0.5$; lower row, $y^{*}\approx 0.1$.

In this last subsection, we address the issue of the influence of azimuthal confinement/extension on the lower transition threshold $Re_{g}$, as the estimations from Figure 3 suggest. In Ref. [36], a similar trend was noted (from measurements in shorter and narrower domains). The mechanism suggested in this former work addressed the presence of oblique stripes rather than their influence on the value of $Re_{g}$. It was thereafter realized that the phenomenon governing the value of $Re_{g}$, and by extension all statistics of the turbulent fraction, is the way different coherent structures interact together dynamically rather than the shape of such individual structures (although that shape certainly influences the interactions). In analogy with pipe [16,42,43] and channel [44,45], the finite turbulent fraction is the result of a dynamical competition between the proliferation of coherent structures and their tendency to decay in number. The transitional range where $\bar{F_{t}}>0$ is dominated by the splitting of coherent structures, whereas instantaneous relaminarizations become rare. We hence focus on the dynamics of splitting events in two different computational domains, namely those with $L_{\theta }=32\pi$ and 128π. Figure 10 contains zooms on the radial velocity plotted for different values of y=cst surfaces (a different value for each row) and for different times (different columns). In Figure 10, the value of $L_{\theta }$ is fixed to 32π, but the circumference in terms of rθ/h varies according to *r*. The global dynamics of these flows can also been scrutinized in the videos made available as Supplementary Materials. The comparison of different values of *y* is useful to confirm that, for all parameters, the spots remain coherent over the gap even during splitting events.

Lateral splitting events are considered in each of these figures and videos. Because of the different advection velocities in the azimuthal direction, spanwise collisions can occur. During spanwise collisions, usually one of the two spots disappears (see also Ref. [21] for similar observations in pCf). This tends to reduce the turbulent fraction while the other surviving spot is still active. In the presence of a short enough spanwise periodicity, a spot collides with itself rather than with a different neighbor. In such periodic domains, the local relaminarization of one spot is equivalent to the extinction of an infinity of identical spots. Hence, the turbulent fraction decreases more than in large domains where individual spots behave more like independent entities. We thus expect more turbulence to proliferate more for larger $L_{\theta }$. As a consequence, the critical Reynolds number $Re_{g}$, for which the rate of proliferation balances the probability to relaminarize locally, is lowered when $L_{\theta }$ is increased, consistently with the thresholds reported in Figure 3 and Table 2. This effect is more marked at lower η.

## 4. Conclusions

The present DNS study deals with the statistical aspects of the intermittent transitional regime of aCf, with an emphasis on the low values of the radius ratio η close to 0.1. It is an extension of the simulations reported recently by Ref. [36]. The paper compares two computational situations, respectively the case of a realistic geometry and the one where the azimuthal extent is larger than the original value of 2π. In Ref. [36], this parametric trick was introduced in an explicit attempt to decouple the effects of wall curvature effects from the effects of azimuthal confinement induced by the geometry. The main conclusion for large η was that the reported absence of oblique laminar-turbulent patterns was due to azimuthal confinement, since they could re-appear for $L_{\theta }>2\pi$. In the present article, the same trick is introduced for η=0.1; however, larger values of $L_{\theta }$ have been tried up to 128π (i.e., 64 times the original value). The oblique patterns do not reappear and a new percolating regime takes place with shorter spatial correlations. The statistical analysis of the STI is convergent as $L_{\theta }$ grows, and is consistent with (1 + 1)-D DP. This updates the results of Ref. [36] where (2 + 1)-D DP was suggested from fits with $L_{\theta }=16\pi$. The present results suggest now that the $L_{\theta }=16\pi$ algebraic statistics was still far from the true thermodynamic limit, while $L_{\theta }=128\pi$ seems to yield more decent results.

To our knowledge, there has been only poor evidence for the cross-over from exponential to algebraic scaling in the shear flow literature, as far as well-resolved simulations of the Navier–Stokes equations are concerned [2]. An exception is the work by Shi et al. [46] in a tilted periodic domain of pCf, which again is not a fully realistic numerical domain. It is interesting to speculate how much the present results can teach us something about a fully realistic system such as cylindrical pipe flow. Naive homotopy of the turbulent regimes is ruled out because of the singularity near the centerline. Instead, we can compare the rate at which these two effectively one-dimensional percolating systems tend towards their own thermodynamic limit. This issue was raised recently in the experimental study by Mukund and Hof [19]. There, despite pipes as long as 3000 diameters, no critical regime (with power-law statistics) was identified, only classical STI as reported in Refs. [47,48]. This issue was attributed to the narrowness of the critical range, and to a clustering property of puffs which delays the convergence to the thermodynamic limit. Here, in aCf with η=0.1, the situation is different but depends on this artificial parameter $L_{\theta }$. To our surprise, power-law statistics of the turbulent fraction as well as of the laminar gap distributions do appear in our simulations as $Re_{w}$ is reduced. All cases shown in Figure 8 suggest a cross-over from turbulent to power-law behavior as $Re_{w}$ is within ≈1% of the critical point. For $L_{\theta }=2\pi$ or around, the turbulent fraction curve still suggests an unconverged power-law. For $L_{\theta }=32\pi$ or 128π, power-law statistics of $\bar{F_{t}}$ are fully consistent with one-dimensional DP appear. This occurs despite a value of $L_{x}$ of only 512*h*, i.e., much less than the pipe flow case and even less if one counts in outer pipe diameters. A possible interpretation is that azimuthal extension, by modifying the interaction with neighboring spots, can suppress the tendency to form clusters, and hence converge faster towards the thermodynamic limit. This is consistent with lower transition thresholds in $Re_{w}$ as well. One is left wondering if a similar approach to cylindrical pipe flow could also easily yield the percolation exponents from simulation measurements.

We conclude by noting that artificially modifying both the shape of turbulent patches and their interaction, as done here using azimuthal extension, is more than an esoteric thought experiment or an exotic parameter study. It is used here as a legitimate strategy in order to untangle complex phenomena, e.g., to decouple confinement from curvature effects. As demonstrated in our recent work using a simple modeling approach [49,50], wall roughness can have similar effects on transitional flows and change the way turbulence invades laminar flows. We expect similar strategies of artificial domain extension to be relevant to such cases too.

## Figures and Tables

**Table 1:** Computational conditions. $L_{i}^{*}$: length of the computational domain in the direction *i*, non-dimensionalized by the gap width $h=(r_{out}-r_{in})$; $L_{out}^{*}$ (resp. $L_{in}^{*}$) the circumference of the outer (resp. inner) cylinder surfaces, normalized by *h*; $N_{i}$: the number of grids.

$\eta =r_{in}/r_{out}$	0.1			0.15	0.2	0.3	
$L_{x}^{*}\times L_{r}^{*}$	512×1			409.6×1			
$L_{\theta }$	2π	32π	128π	128π	112π	2π	96π
$L_{out}^{*}\;\left(=L_{\theta }\;r_{out}^{*}\right)$	7.0	111.7	446.8	473.1	439.8	9.0	430.8
$L_{in}^{*}\;\left(=L_{\theta }\;r_{in}^{*}\right)$	0.7	11.2	44.7	71.0	88.0	2.7	129.2
$N_{x}\times N_{r}$	2048×64						
$N_{\theta }$	32	512	2048	2048	2048	64	2048

**Table 2:** Critical Reynolds number $Re_{g}$ and critical exponent β depending on geometrical parameters η (radius ratio) and $L_{\theta }$ (azimuthal extension). In addition, shown is the fitting range to estimate $Re_{g}$ and β. ${}^{†}$: not measured.

$\eta =r_{\mathrm{in}}/r_{\mathrm{out}}$	$L_{\theta }$	Fitting Range	Reg	β
0.10	2π	407.5–460.0	406.9	0.31(3)
0.10	32π	277.5–300.0	269.0	0.26(2)
0.10	128π	263.0–270.0	261.7	0.28(2)
0.15	128π	— ${}^{†}$	290.5	— ${}^{†}$
0.20	112π	— ${}^{†}$	303.5	— ${}^{†}$
0.30	2π	389.0–395.0	388.7	0.12(2)
0.30	96π	320.5–375.0	319.0	0.28(1)
(1 + 1)-D DP model		—	—	0.276

## Supplementary Materials

Video S1: Time evolution of turbulent fraction $F_{t}\left(t\right)$ and of fluctuating velocity fields visualized at mid-gap, for η=0.1 with an artificially extended azimuthal domain size of $L_{\theta }=128\pi$. On the right column, contours show *x*-θ distributions of the radial velocity fluctuation $u_{r}^{\prime }$ normalized by the inner-cylinder velocity $U_{w}$. Top (orange box and curve in the graph): above the global critical Reynolds number $Re_{g}$. Middle (red): near $Re_{g}$. Bottom (black): below $Re_{g}$. A supporting video article is available at https://doi.org/10.5281/zenodo.3985963.

## Abbreviations

The following abbreviations are used in this manuscript:

aCf	annular Couette flow
CDF	cumulative distribution function
DNS	direct numerical simulation
DP	direct percolation
pCf	plane Couette flow
pPf	plane Poiseuille flow
rms	root-mean-square value
STI	spatiotemporal intermittency
